# 17-Allylamino-17-demethoxygeldanamycin induces downregulation of critical Hsp90 protein clients and results in cell cycle arrest and apoptosis of human urinary bladder cancer cells

**DOI:** 10.1186/1471-2407-10-481

**Published:** 2010-09-09

**Authors:** Panagiotis K Karkoulis, Dimitrios J Stravopodis, Lukas H Margaritis, Gerassimos E Voutsinas

**Affiliations:** 1 Laboratory of Environmental Mutagenesis and Carcinogenesis, Institute of Biology, National Center for Scientific Research (NCSR) "Demokritos", 15310 Athens, Greece; 2 Department of Cell Biology and Biophysics, Faculty of Biology, University of Athens, Panepistimiopolis, Zografou, 15784 Athens, Greece; 3 Institute of Biology, NCSR "Demokritos", 15310 Athens, Greece

## Abstract

**Background:**

17-Allylamino-17-demethoxygeldanamycin (17-AAG), a benzoquinone ansamycin antibiotic, specifically targets heat shock protein 90 (Hsp90) and interferes with its function as a molecular chaperone that maintains the structural and functional integrity of various protein clients involved in cellular signaling. In this study, we have investigated the effect of 17-AAG on the regulation of Hsp90-dependent signaling pathways directly implicated in cell cycle progression, survival and motility of human urinary bladder cancer cell lines.

**Methods:**

We have used MTT-based assays, FACS analysis, Western blotting, semi-quantitative RT-PCR, immunocytochemistry and scratch-wound assay in RT4, RT112 and T24 human urinary bladder cancer cell lines.

**Results:**

We have demonstrated that, upon 17-AAG treatment, bladder cancer cells are arrested in the G1 phase of the cell cycle and eventually undergo apoptotic cell death in a dose-dependent manner. Furthermore, 17-AAG administration was shown to induce a pronounced downregulation of multiple Hsp90 protein clients and other downstream effectors, such as IGF-IR, Akt, IKK-α, IKK-β, FOXO1, ERK1/2 and c-Met, resulting in sequestration-mediated inactivation of NF-κB, reduced cell proliferation and decline of cell motility.

**Conclusions:**

In total, we have clearly evinced a dose-dependent and cell type-specific effect of 17-AAG on cell cycle progression, survival and motility of human bladder cancer cells, due to downregulation of multiple Hsp90 clients and subsequent disruption of signaling integrity.

## Background

Urinary bladder cancer is the fifth most common malignancy in the industrialized world and the second most frequent malignancy of the genitourinary tract, demonstrating high heterogeneity and differential response to clinical treatment [1,2]. Bladder cancer incidence, morbidity and mortality rates vary by genetic background, country, gender and age [3]. The most prevalent type of bladder cancer in the developed world is urothelial carcinoma (UC), representing over 90% of all bladder cancers, followed by squamous cell carcinoma (5%) and adenocarcinoma (2%) [4]. A high percentage of bladder cancer patients (20-30%) present with an aggressive muscle-invasive tumor of low differentiation, whereas the rest develop superficial, highly differentiated, non-invasive papillary tumors, 30% of which, nevertheless, are estimated to recur to invasive. Unfortunately, more than half of the patients with invasive tumors will develop distant metastases over a time period of two years [5], while the five-year survival rate for metastatic disease is as low as 6%. This apparent heterogeneity in bladder cancer is thought to be mainly due to discrete genetic alterations involved in tumor development and progression. Thus, since established systemic chemotherapy protocols for metastatic urothelial carcinoma are associated with significant toxicities, new clinical protocols designed for higher efficiency, while reducing the adverse side effects, are urgently needed.

Relatively recently, heat shock protein 90 (Hsp90) has emerged as an important target in cancer therapy. Hsp90 normally accounts for approximately 1-2% of the total cytosolic protein content, while under stress conditions, its levels increase up to 4-6% of the whole proteomic load of the cell [6-8]. The Hsp90 chaperone activity relies on its transient NH_2_-terminal dimerization, which facilitates its intrinsic ATPase activity [9]. The Hsp90 chaperone complex maintains the correct folding, cellular localization and activity of a broad range of protein clients that are implicated in various signal transduction pathways involved, among others, in cell proliferation, differentiation and survival [7,10]. There is evidence that Hsp90 is a major facilitator of cellular response to extracellular signals, particularly required for normal cell growth, proliferation and development [11]. On the other hand, over-expression and/or presence of mutations in a variety of Hsp90 protein clients during cancer initiation is associated with a requirement for increased Hsp90 levels in order to maintain the active conformations and thus functional integrities of these oncogenic molecules. In this frame, Hsp90 is a key molecule in the conformational maturation of several *bona fide *oncogenic signaling proteins, such as HER2/ErbB2, Akt, Met, Raf1, p53 and HIF-1α [10,12]. Therefore, due to the dependence of cancer cells upon specific Hsp90 oncogenic protein clients, inhibition of Hsp90 was shown to be able to negatively interfere with a number of important signaling pathways involved in cell development, proliferation, survival and motility, arousing significant interest in the field of cancer therapeutics [13].

Thus, a diverse group of molecules that target Hsp90 have been discovered or synthesized over the past several years. These include natural products, such as geldanamycin, radicicol and derivatives; synthetic purine-based inhibitors, such as PU3, PU24FCI and PU29FCI; and compounds that bind to Hsp90 on a secondary ATP-binding site, such as novobiocin and cisplatin [6]. The geldanamycin derivative 17-allylamino-17-demethoxygeldanamycin (17-AAG) possesses an allylamino group at position 17 of the scaffold structure of geldanamycin [6]. Compared to the parental compound, 17-AAG demonstrates reduced toxicity, with enhanced biological activity and metabolic stability, retaining the Hsp90-related therapeutic characteristics. 17-AAG exerts its anti-tumor potency through its high affinity binding to the NH_2_-terminal ATP-interacting domain of Hsp90, thus inhibiting its ability to form transient, active homodimers, and to consequently participate in chaperone-client complexes, with a subsequent hindering of client maturation and stabilization.

In this context, here, we have thoroughly studied the effects of Hsp90 inhibition by 17-AAG on the Hsp90-assisted signaling repertoire associated with cell cycle progression, apoptosis and motility in three human urinary bladder cancer cell lines of different malignancy grade, namely RT4 (grade I), RT112 (grade I-II) and T24 (grade III).

## Methods

### Drugs and reagents

17-AAG chemotherapeutic reagent was obtained from InvivoGen (San Diego, California, USA). Polyclonal and monoclonal antibodies against Caspase-8, Caspase-9, Caspase-3, PARP, Lamin A/C, phospho-Akt (Ser473), phospho-Akt (Thr308), Akt, phospho-IGF-ΙRβ (Tyr1131), IGF-ΙRα, FOXO, phospho-FOXO, phospho-IKKα/β (Ser180/Ser181), IKKα, IKKβ, phospho-p44/42 (Thr202/Tyr204), p44/42, α-tubulin, phospho-c-Met (Tyr1234/Tyr1235), c-Met, CHIP and pan-actin were purchased from Cell Signaling Technology Inc. (Hertfordshire, UK), whereas antibodies against Hsp90α/β, Hsp70, Cdk4, pRb, E2F1 and NF-κB (p65) were supplied by Santa Cruz Biotechnology Inc. (California, USA). Enhanced Chemilluminescence (ECL) Western blot detection reagents were obtained from GE Healthcare Life Sciences (Buckinghamshire, UK). Oligonucleotide primers were synthesized by Operon (California, USA). All other chemicals were of analytical grade from Sigma-Aldrich (Missouri, USA), Fluka (Hannover, Germany) and AppliChem GmbH (Darmstadt, Germany).

### Cell lines and culture conditions

The present study was performed on three human urinary bladder cancer cell lines, namely RT4, RT112 and T24, all originating from urothelial carcinomas. RT4 cells are derived from grade I tumor and were obtained from the European Collection of Animal Cell Cultures (Salisbury, UK); RT112 cells are derived from a grade I→II tumor, whereas T24 cells are derived from a grade III tumor. RT112 and T24 cells were a generous gift from Professor J. R. Masters (Prostate Cancer Research Centre, Institute of Urology, University College London, UK). Cells were maintained in DMEM, supplemented with 10% heat inactivated FBS, at 37°C in a humidified 5% CO_2 _atmosphere. All cell culture media and reagents were supplied by Biochrom AG (Berlin, Germany).

### Cell viability assay

Urinary bladder cancer cells were seeded at a density of 15-20 × 10^3 ^per well into 48-well plates and treated with various drug concentrations for 24 h. The next day, cells were incubated in methylthiazole tetrazolium (MTT) solution. The spectrophotometric absorbance was measured in an ELISA microtiter plate reader (Dynatech MR5000, Dynatech Laboratories, Virginia, USA) at 550 nm, using measurement at 630 nm as reference. Absorbance rates obtained by untreated cells were considered as 100% cell survival. Each assay was repeated at least three times, using three wells per drug concentration in each experimental condition.

### Cell cycle analysis

Bladder cancer cells were seeded at a density of approximately 5 × 10^5 ^in 100 mm plates and drug treatments of different concentrations of 17-AAG were applied for 24 h. Cells were collected, fixed in 1% methanol-free formaldehyde for 20 min and subsequently suspended in a 70% ethanol solution and stored at -20°C to dehydrate. Twenty four hours later, cells were suspended in 1 ml of 0.1% Triton X-100 solution and incubated in 500 μl of propidium iodide solution (50 μg/ml) containing 250 μg of DNase-free RNase A. Cells were analyzed with a Beckton Dickinson's FACScalibur (California, USA) at 542 nm and results were processed with the Modfit software program. Each assay was repeated three times.

### Immunoblotting

Whole cell protein extracts were prepared as previously described [14]. Approximately 30 μg of total protein preparations were resolved by SDS-polyacrylamide gel electrophoresis and subsequently electro-transferred overnight onto nitrocellulose membranes of 0.45 μm pore size (Schleicher and Schuell GmbH, Dassel, Germany). Membrane blocking was performed in TBS-T (20 mM Tris-HCl, pH 7.6, 137 mM NaCl and 0.1% Tween-20) containing 5% non-fat dry milk (or 5% BSA grade V, where appropriate) and membranes were incubated with the appropriate antibodies at room temperature for 90 min, followed by an overnight incubation at 4°C. The next day, membranes were incubated with the suitable anti-mouse or anti-rabbit HRP-conjugated secondary antibody and immunoreacting proteins were detected using an ECL Western blotting kit according to the manufacturer's instructions. All immunoblotting experiments were repeated three times.

### RT-PCR analysis

Total RNA from both control and treated cells was extracted as previously described [14]. 1 μl of cDNA solution was amplified by PCR in a total volume of 25 μl, using cDNA-specific primers corresponding to the various mRNA species examined in the present study. Most gene-specific cDNA primer sequences and associated PCR information have been previously described [14,15], whereas additional genes amplified for the purpose of this study were: *Cyclin D1 *(forward: 5'-GTGTCCTACTTCAAATGTGTGC-3', reverse: 5'-GGAGTTGTCGGTGTAGATGC-3', Ta: 57°C, 30 cycles), *Hsp90α *(forward: 5'-CCAAGATGCCTGAGGAAAC-3', reverse: 5'-TCATACCGGATTTTGTCCAAT-3' Ta: 53°C, 30 cycles) and *Hsp90β *(forward: 5'-TCCTTTTCTTTTCAAGATGCC-3', reverse: 5'-TGTCCAACTTCGAAGGGTCT-3', Ta: 54°C, 30 cycles). The obtained PCR fragments were resolved in 2% agarose gels, according to standard procedures. All RT-PCR experiments were repeated three times.

### Immunofluorescence

T24 urinary bladder cancer cells were seeded on poly-L-lysine coated slides (Thermo Fisher Scientific Inc., Minnesota, USA) and treated with a 17-AAG concentration of 10 μM for 24 h. After treatment, slides were fixed with a paraformaldehyde solution (3% in 1 × PBS) for 15 min at room temperature. Cell permeabilization was achieved by administration of a Triton X-100 solution (0.5% in 1 × PBS) for 20 min. Subsequently, slides were blocked with a 1% BSA solution for 60 min and then incubated with an NF-κB anti-p65 antibody overnight at 4°C. The next day, slides were incubated with a FITC-conjugated anti-rabbit secondary antibody (Jackson ImmunoResearch Laboratories Inc., Pennsylvania, USA), while nuclear staining of cells was obtained by incubation with propidium iodide solution (1 μg/ml in 1 × PBS containing RNase A). Finally, cells were observed under a Nikon EZ-C1 confocal microscope (Nikon Instruments Inc., Japan). Images taken were processed with the support of the Nikon EZ-C1 software program. Immunofluorescence experiments were repeated three times.

### Scratch-wound assay

Human urinary bladder cancer cells were seeded at a density of 5 × 10^5 ^per 100 mm diameter Petri dish and incubated overnight. The day after, the surfaces of the dishes were mildly scratched with a sterile Pasteur pipette and images were taken under a Carl Zeiss Axiovert 25 (Thornwood New York, USA) inverted microscope with the use of a Cannon Powershot G9 digital camera and a PS-Remote software program. Then, cells were treated with a 10 μΜ 17-AAG solution and incubated overnight at 37°C in a humidified 5% CO_2 _atmosphere. Twenty four hours later, treated and untreated cells were observed under the inverted microscope at the scratch-wounded areas. Scratch-wound assays were repeated three times.

## Results

**Figure 1:**
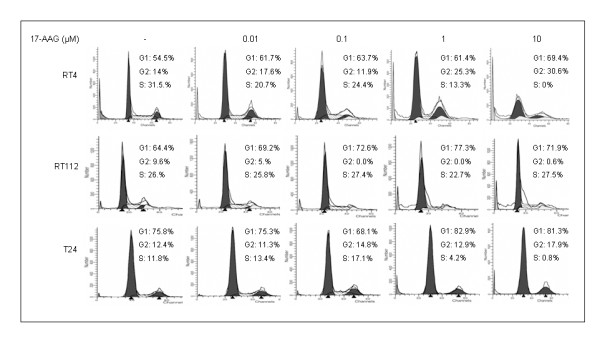
**Cell cycle progression analysis of human bladder cancer cells treated with 17-AAG for 24 hours**. Representative FACS analysis of RT4, RT112 and T24 bladder cancer cells and percentage of total cell content in distinct phases of the mitotic course are displayed. The cell cycle experiments were repeated three times.

*17-AAG displays an inhibitory effect on cell cycle progression of human urinary bladder cancer cells*. We have studied the effect of 24-hours 17-AAG treatment on the course of the cell cycle of RT4, RT112 and T24 human urinary bladder cancer cells using flow cytometry (Figure 1). As presented herein, RT4 cancer cells displayed a G1 arrest (from 54.5% in the control to 69.4% at 10 μM), while a G2-phase inhibition was also observed (from 14% in the control to 30.6% at 10 μM), both at higher concentrations of the drug. Upon treatment with 17-AAG, RT112 presented an increase in the percentage of cells accumulating in G1 phase (from 64.4% in the control reaching a peak value of 77.3% at the 1 μΜ dosage), accompanied by a possible S block, since the percentage of cells in the S phase remained practically unchanged (from 26% to 27.5%), whereas the number of cells in phase G2 was constantly decreasing (from 9.6% to 0%). Treatment of T24 cells with 17-AAG was able to induce a moderate G1 block (from 75.8% in the control to 81.3% at 10 μΜ), while it was also found to cause an additional mild arrest of the cell cycle in phase G2 (from 12.4% in the control to 17.9% at 10 μΜ).

**Figure 2:**
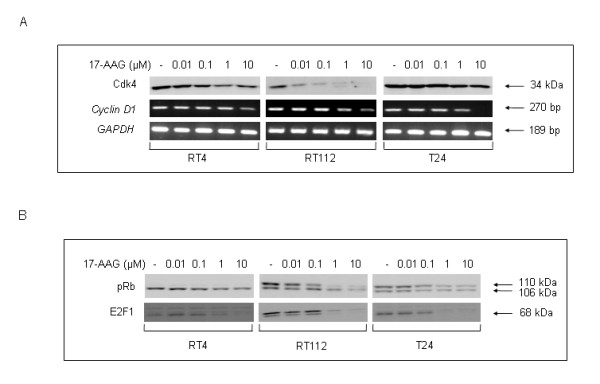
**17-AAG-induced G1 cell cycle block is due to downregulation of specific Hsp90 clients.** (A) Detection of Cdk4 protein (Western blotting) and *Cyclin D1 *gene (RT-PCR), both critically implicated in the G1 to S phase transition, over a 24-hours treatment period of human bladder cancer cells with 17-AAG. The mRNA expression levels of the internal control gene *GAPDH *were also quantified by an RT-PCR approach. (B) Western blotting of the cell cycle regulatory proteins pRb and E2F1, after exposure of RT4, RT112 and T24 cells to different 17-AAG doses. Immunoblotting and RT-PCR experiments were repeated three times.

In order to further illuminate the G1 block observed, we examined the effect of 17-AAG on Cyclin/Cyclin-dependent kinase (Cdk) complex components, which assist dividing cells to overcome the G1-phase checkpoint (Figure 2A). We have found that Cdk4 protein levels display a dose-dependent and cell type-specific decrease, with Cdk4 downregulation being more prominent in RT112 than RT4 cells, whereas in T24 this was kept to a minimum. A similar pattern of downregulation in all the cell lines was demonstrated when studying the expression levels of *Cyclin D1 *mRNA, with T24 exposed to the higher drug dose manifesting the most severe response, hence suggesting a possible Cyclin D1 and Cdk4 involvement in the observed 17-AAG-induced G1 cell cycle block (Figure 2A). Furthermore, the expression and activation of other downstream significant modulators of cell cycle progression, such as pRb protein and its interacting partner transcription factor E2F1 were examined (Figure 2B). After treatment with 17-AAG, pRb protein levels were shown to display a dose-dependent downregulation in all three cell lines examined in this study. Interestingly, in RT4 cells pRb protein is not phosphorylated either in the control or after drug exposure, whereas phosphorylation in RT112 and T24 was found to decrease with increasing 17-AAG doses, in a cell type-specific manner. In relation to this, E2F1 protein levels also displayed a clear downregulation pattern in all three cell lines, rendering this transcription factor practically undetectable in the higher doses (1 and 10 μM), also suggesting a possible E2F1 involvement in the observed 17-AAG-induced G1 cell cycle block.

**Figure 3:**
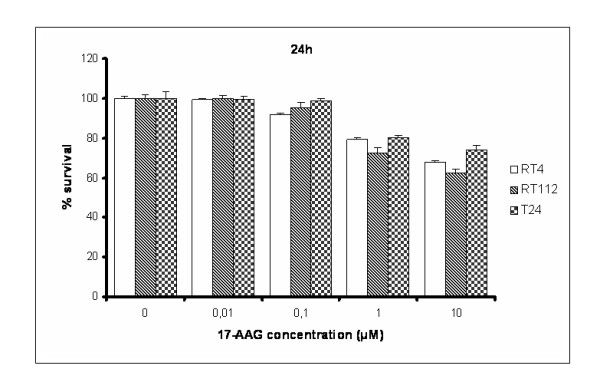
**MTT cytotoxicity assays performed on RT4, RT112 and T24 bladder cancer cells, upon 24 hours of treatment with 17-AAG**. All assays were implemented three times, while the obtained standard deviations (error bars) are depicted on the top of each value bar.

**Figure 4:**
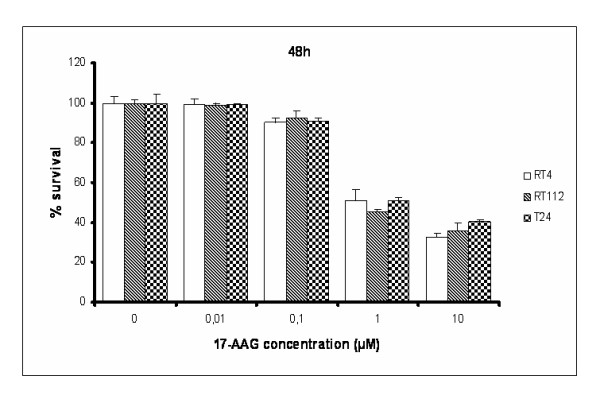
**MTT cytotoxicity assays performed on RT4, RT112 and T24 bladder cancer cells, upon 48 hours of treatment with 17-AAG**. All assays were implemented three times, while the obtained standard deviations (error bars) are depicted on the top of each value bar.

*17-AAG manifests a cytotoxic effect on human bladder cancer cell lines*. To assess the biological effect of 17-AAG on bladder cancer cell survival, we performed MTT assays on RT4, RT112 and T24 cells, incubated with increasing concentrations of the drug for 24 and 48 hours (Figures 3 and 4). All three cell lines showed a dose-dependent decrease in cell viability. It seems that RT112 cells are more sensitive than RT4 to the cytotoxic activity of 17-AAG after 24 hours of treatment, while T24 are slightly more resistant. Significant numbers of cells, alive but committed to apoptosis at 24 hours, were dead after 48-hours treatment, so the percentage in cell survival was dramatically decreased and almost equal in all three bladder cancer cell lines.

**Figure 5:**
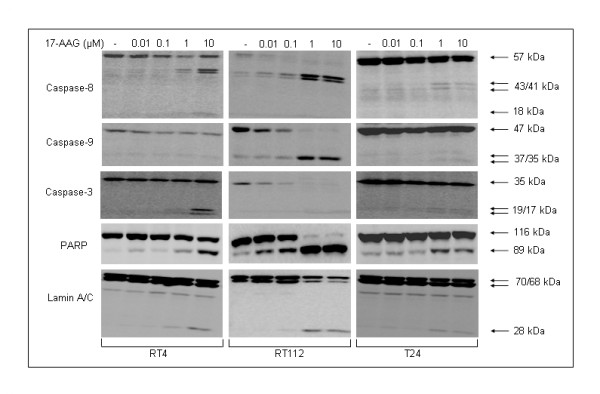
**17-AAG activates Caspase-dependent death processes in bladder cancer cells.** Apoptotic death induced by 17-AAG administration in human urinary bladder cancer cells was characterized by reduced expression and proteolytic cleavage profiles of apoptosis-related proteins, in response to various drug concentrations, for 24-hours exposure period. Treatment of RT4, RT112 and T24 cells with 17-AAG resulted in prominent cleavage and activation of critical members of the Caspase apoptotic machinery in all three cell lines, along with notable proteolytic processing of the Caspase repertoire characteristic substrates PARP and Lamin A/C. All apoptosis immunoblotting experiments were repeated three times.

*17-AAG induces activation of Caspase-dependent death processes in bladder cancer cells*. 17-AAG-induced reduction of cell survival was found to be associated with proteolytic cleavage of critical members of the Caspase family (Caspase-8, Caspase-9 and Caspase-3) and characteristics of apoptotic death. Protein expression levels and activation of Caspase proteases are shown in Figure 5. The downregulation patterns of precursor Caspase protein levels were more pronounced in RT112 cells, combined with dose-dependent increases in proteolytic cleavage products of Caspase-8 (43, 41 and 18 kDa), Caspase-9 (37 and 35 kDa) and Caspase-3 (19 and 17 kDa). These data clearly demonstrate the ability of 17-AAG to induce Caspase-dependent death in all three bladder cancer cell lines studied here. This fact was further certified by the detection of intense cleavage of the Caspase repertoire substrates PARP and Lamin A/C upon administration of relatively high concentrations of 17-AAG (1 and 10 μΜ) in RT4, RT112 and T24 bladder cancer cell lines.

**Figure 6:**
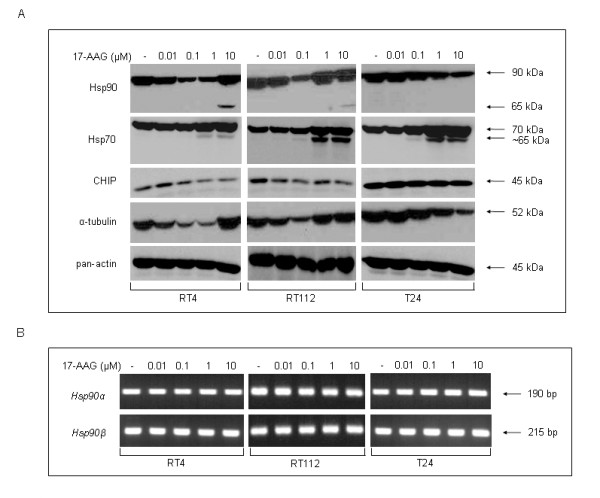
**17-AAG-induced downregulation of Hsp90 in urinary bladder cancer cells, after 24 hours of drug treatment**. (Α) Total and truncated protein levels of critical members of the "chaperosome", such as Hsp90, Hsp70 and CHIP, are visualized by a Western blotting approach. Pan-actin was used as protein of reference, while α-tubulin revealed a similar to Hsp90 expression profile. The transcriptional activity analysis of *Hsp90 *genes (*α *and *β*), in response to 17-AAG, is provided in (B). All experiments were repeated three times.

*Exposure of human bladder cancer cells to 17-AAG results in downregulation of Hsp90*. The effect of 17-AAG administration on Hsp90 and co-chaperone Hsp70 structural integrities in RT4, RT112 and T24 bladder cancer cells was examined by western immunoblotting (Figure 6A). In RT4 cells, 24 h incubation with 17-AAG resulted in a dose-dependent reduction of Hsp90 protein levels, up to the concentration of 1 μΜ. Intriguingly, at the highest dose of 10 μΜ, the levels of Hsp90 protein rose again significantly, disrupting the downregulation pattern, whereas an Hsp90 cleavage product with a molecular weight of approximately 65 kDa was generated. The same pattern of initial reduction (lower doses) and following increase (higher doses) of total Hsp90 protein levels was observed in RT112 cells as well, only this was found to occur at even lower doses. More specifically, RT112 cells displayed maximum Hsp90 downregulation at the dose of 0.1 μM 17-AAG, whereas a significant upregulation of total cellular Hsp90 levels was observed at 1 and 10 μM of 17-AAG, with production of the Hsp90 cleavage fragment at the highest drug dose (10 μM). This pattern could not be detected in the malignant cell line T24, where the levels of Hsp90 proved to follow a consistent dose-dependent decrease. Regarding the protein levels of Hsp70 co-chaperone, these appear to follow a dose-dependent increase, which becomes quite significant in response to relatively high doses of 17-AAG (1 and 10 μΜ) in all three cell lines. In addition, at the two (RT4) or three (RT112 and T24) higher doses of the drug (0.1, 1 and 10 μM), we were able to detect the presence of an Hsp70 protein cleavage product with a molecular weight of approximately 65 kDa, which also seemed to display a cell type-specific and dose-dependent formation pattern.

In order to study the cause of the 17-AAG-induced response pattern of Hsp90 in the three cell lines, we decided to analyze the expression of another member of the Hsp90 chaperone complex, namely Carboxyl terminus of Hsp70 interacting protein (CHIP), an E3 ubiquitin ligase, which regulates the turnover of Hsp90 protein clients in mammalian cells, but also Hsp90 itself, via ubiquitination of specific residues of the chaperone, therefore making it a suitable candidate for proteasomal degradation [16-18]. In the bladder cancer cell lines used in this study, CHIP showed a dose-dependent and cell type-specific decrease in response to 17-AAG administration, with RT4 and RT112 cells exhibiting the most notable reduction, whereas CHIP protein control levels were found to steadily increase from RT4 to RT112 and then T24 cells. Pan-actin was used as protein of reference in all experiments performed herein, whereas α-tubulin, interestingly, appeared to follow an expression pattern very similar to that of Hsp90. This is consistent with the recently discovered association between tubulin and the Hsp90 chaperone complex [19].

Finally, we examined the transcriptional profiles of *Hsp90α *and *Hsp90β *genes in response to the drug, in order to identify a possible association of 17-AAG-induced Hsp90 downregulation with transcriptional repression of *Hsp90 *genes. In this frame, *Hsp90 *mRNA expression levels were tested and found to remain unaffected in all the cell lines used here (Figure 6B), thus excluding any type of transcriptional control involvement in the 17-AAG-induced downregulation of Hsp90 protein.

**Figure 7:**
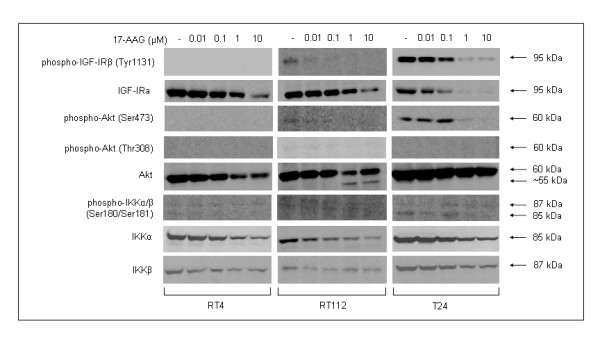
**17-AAG administration results in functional attenuation of Akt signaling in bladder cancer cells**. Inhibition of Hsp90 leads to prominent downregulation of the Akt-dependent signaling pathway, presenting with pronounced decrease of constitutively phosphorylated, and therefore activated, IGF-I receptor, Akt kinase and downstream signaling components, such as members of the IKK family (IKKα and IKKβ). All experiments were repeated three times.

*17-AAG administration leads to downregulation of critical targets in the IGF-IR/Akt signaling pathway and results in NF-κB inactivation*. Upon exposure to 17-AAG, a variety of Hsp90 protein clients, mainly kinases and transcription factors, were shown to be notably downregulated in the human urinary bladder cancer cell lines used in this study (Figure 7). In response to the drug, the total protein levels of IGF-I receptor (IGF-IR) showed a prominent dose-dependent reduction in all three bladder cancer cell lines, with T24 exhibiting the most potent effect (Figure 7). Remarkably, even though in RT112 and T24 cells the phosphorylated receptor levels, albeit weak in the former and strong in the latter, were similarly found to decrease in a dose-dependent manner, in RT4 cells no phosphorylation of the IGF-IR protein could ever be detected.

A prominent downstream target of the IGF-I receptor and distinguished protein member of the Hsp90 clientele is the Akt kinase, a critical regulatory component in many signaling pathways. Upon administration of 17-AAG, Akt proved to be downregulated in all three bladder cancer cell lines, in a dose-dependent manner (Figure 7). Interestingly, in RT112 cells we were able to observe the formation of a lower molecular weight fragment, possibly representing a cleavage product of the intact Akt kinase (55 kDa, approximately), specifically generated after exposure to 1 and 10 μM 17-AAG. Next, we tested the presence of phosphorylated Akt in the same cells before and after 17-AAG treatment. Even though phosphorylation of Akt on serine residue at position 473 (Ser473) could not be detected in RT4, it was marginal in RT112, and highly activated in T24 cells. In RT112 and T24 cell lines, Akt phosphorylation showed a dose-dependent decrease, resulting in an almost total elimination of the active form of the protein from drug concentrations higher than 0.1 μM for RT112 and 1 μM for T24 cells. Akt phosphorylation on threonine residue at position 308 (Thr308) ranged from absent (RT4 and T24) to marginal (RT112) levels.

**Figure 8:**
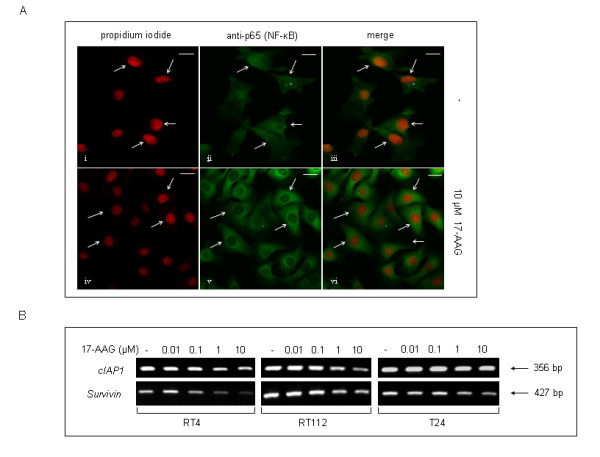
**NF-κB functional impairment, in response to 17-AAG administration in bladder cancer cells.** (A) Characteristic immunofluorescence images of T24 cells presenting NF-κB (green color) cellular localization, under control (i-iii) or 10 μM of 17-AAG (iv-vi) treatment conditions (scale bars: 10 μm). Nuclear counterstaining was performed by the utilization of propidium iodide, while images were visualized by Confocal microscopy and appropriately merged to reveal NF-κB localization in bladder cancer cells (white arrows). (B) Expression profiles of the two anti-apoptotic NF-κB target genes *cIAP1 *and *Survivin *in RT4, RT112 and T24 bladder cancer cells, upon exposure to 17-AAG for 24 hours. All experiments were repeated three times.

17-AAG-induced Akt functional repression and degradation was accompanied by expression level reduction of the downstream targets ΙΚΚα and IKKβ, which clearly exhibit a dose-dependent downregulation pattern consistent with their status as *bona fide *Hsp90 chaperone clients. Moreover, the activated forms of IKKα and IKKβ kinases, phosphorylated on serine residues at positions 180 and 181, respectively, were detected at very low levels in all three cell lines (with T24 revealing the strongest expression profile), exhibiting a dose-dependent inhibition in response to 17-AAG administration. Furthermore, the combinational inhibitory effect of 17-AAG on key molecules of the IGF-IR/Akt/IKK axis was found to induce inactivation of NF-κB transcription factor, a downstream target of this pathway, ultimately resulting in its relocation to the cytoplasm, therefore rendering it unable to exert regulatory control upon a vast number of genes involved in cell proliferation and survival. As illustrated in Figure 8A, it is clear that 17-AAG promotes NF-κB inactivation in T24 bladder cancer cells due to nuclear exclusion of the factor, in contrast to the compartmentalization profile observed in control cells, where NF-κB is located both inside the nucleus and the cytoplasm. To reinforce our findings on 17-AAG-induced NF-κB inhibition in bladder cancer cells, the mRNA expression of two representative anti-apoptotic NF-κB target genes, namely *cIAP1 *and *Survivin*, was examined using an RT-PCR approach (Figure 8B). Thus, in response to 17-AAG, both genes were found to be downregulated in a cell type-specific and dose-dependent manner, with RT4 and RT112 cells displaying stronger reductions of mRNA levels compared to the malignant T24 ones.

**Figure 9:**
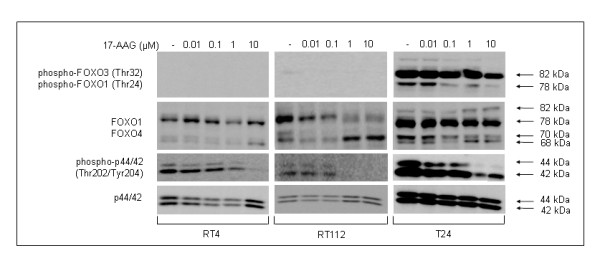
**17-AAG induces downregulation of FOXO and ERK1/2 signaling mediators in bladder cancer cells.** Detection of total and constitutively phosphorylated protein levels of FOXO transcription factors and Erk1/2 (p44/42) kinases in all three bladder cancer cell lines, upon 17-AAG administration for 24 hours, via a Western blotting approach. All immunoblotting experiments were repeated three times.

In addition, we examined one critical group of Akt downstream targets tightly associated with cell death inhibition signaling, the Forkhead family of transcription factors (FOXO: Forkhead box O). As shown in Figure 9, total FOXO1 protein detected at 78 kDa was found to display a characteristic cell type-specific and dose-dependent reduction in response to the drug, which was incomparably prominent in RT112 cells. On the other hand, total FOXO4 protein levels in all three bladder cancer cell lines exhibited an expression pattern similar to the one observed for Hsp90 and α-tubulin (Figure 9, the lowest band of 68 kDa). Furthermore, the phosphorylation status of FOXO proteins was also examined. In RT4 cells, no detectable phosphorylated FOXO transcription factors could be observed, while in RT112 cells only phospho-FOXO3 was traced in the control with subsequent elimination starting from the lowest drug dose. Interestingly, T24 cells were characterized by high levels of phosphorylated FOXO1 and FOXO3 proteins, likely reflecting the low differentiation level of these cells, whereas exposure to 17-AAG resulted in dose-dependent downregulation of both phosphorylated FOXO family members, supporting the induction of dephosphorylation-mediated nuclear sequestration of the Forkhead factors, with subsequent transactivation of apoptotic target genes.

Furthermore, we studied the effect of 17-AAG on the Ras-Raf-MEK-ERK pathway (known to usually cross-talk with Akt) in bladder cancer cells, by detection of total and phosphorylated p44/42 (Erk1/2) kinase protein levels. As illustrated in Figure 9, upon 17-AAG administration, total p44/42 levels in both RT4 and RT112 cell lines exhibited a pattern reminiscent of the one previously encountered in Hsp90, α-tubulin and FOXO4. More precisely, in RT4 cells, p44/42 protein levels displayed a pattern of dose-dependent downregulation up to 1 μM concentration of the drug, whereas a significant increase could be observed in the highest dose (10 μM). In RT112 cells, the pattern was similar, but shifted to lower concentrations. Thus, total p44/42 protein levels were found to manifest a drug-mediated reduction in the lower concentrations only, whereas in the higher ones a clear increase could be observed. On the contrary, in T24 cells, a slight but notable dose-dependent decrease of p44/42 expression levels was observed. In order to evaluate the potency of p44/42 signal transduction upon exposure to 17-AAG, the active form of the protein (phosphorylated on threonine and tyrosine residues at positions 202 and 204, respectively) was analyzed. As shown in Figure 9, all three cell lines demonstrated a severe dose-dependent reduction of active p44/42, therefore causing the downregulation of a variety of downstream targets, mainly involved in cell proliferation and survival. *In toto*, 17-AAG proved to induce a prominent inhibitory effect upon multiple Hsp90 clients, affecting both the NF-κB and the FOXO axes of the IGF-IR/Akt signaling repertoire, as well as the p44/p42-dependent pathway, likely promoting the downregulation of downstream targets and finally leading to decreased cell proliferation and survival.

**Figure 10:**
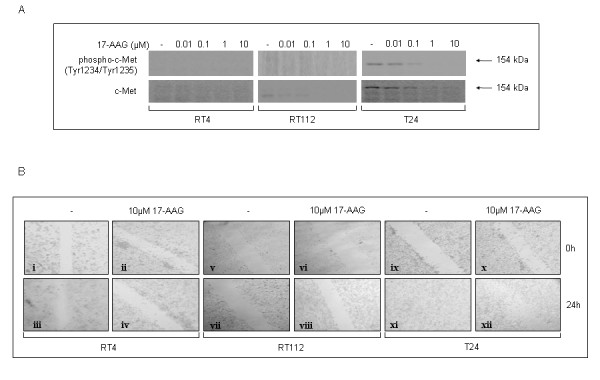
**Exposure to 17-AAG results in reduced cancer cell motility and proliferation**. (A) Western blotting analysis revealing the expression profile of total and phosphorylated c-Met protein in all three bladder cancer cell lines, upon a 24-hours incubation with different concentrations of 17-AAG. (B) Scratch-wound assays conducted on RT4 (i-iv), RT112 (v-viii) and T24 (ix-xii) cells, under control conditions or in the presence of 10 μM 17-AAG. T24 highly malignant cells display strong wound-healing potential (panel xi) over a 24-hours period, compared to RT4 and RT112 (panels iii and vii, respectively) cells, under control conditions. Interestingly, 17-AAG administration induces a moderate impairment of T24 cell motility and proliferative dynamics (panel xii), thus only partly prohibiting, in contrast to RT4 and RT112, the efficient closure of the gap (scratch-wound). Cells were observed under an inverted microscope and pictures were taken at a 20 × magnification. All experiments were repeated three times.

*17-AAG administration reduces urinary bladder cancer cell motility*. Cancer cell motility is an important determinant of epithelial-mesenchymal transition (EMT), which underlies the early phase of the tumor invasion program. One of the key components in EMT-dependent tumor proliferation, cell motility and invasion signaling is the hepatocyte growth factor (HGF) receptor, also referred to as c-Met. In order to assess the effect of 17-AAG on c-Met pathway activation in RT4, RT112 and T24 bladder cancer cell lines, we have examined the total and phosphorylated protein levels of c-Met in response to drug exposure. As shown in Figure 10A, the low malignancy grade RT4 and RT112 cells were characterized by non to slightly detectable total c-Met protein levels, respectively, whereas in grade III T24 cells, c-Met protein could be easily recognized, exhibiting a drug dose-dependent downregulation pattern and reaching a complete elimination by the 1 μM dose of 17-AAG. The phosphorylated, active form of the protein was observed exclusively in high grade T24 cells, displaying a dose-dependent reduction profile, whereas RT4 and RT112 cells presented with undetectable (or hardly detectable) levels of constitutive activation (Figure 10A).

To further illuminate the effect of 17-AAG on the efficiency of bladder cancer cell motility, we have conducted scratch-wound assays on all the cell lines examined herein (Figure 10B). As clearly demonstrated in this work, the low malignancy grade cell lines RT4 and RT112 presented with reduced proliferation and motility potency, unable to heal the "wounds" during a 24-hours incubation period, either under high 17-AAG concentration (10 μΜ) or control conditions (Figure 10B, RT4: panels i-iv, RT112: panels v-viii). In contrast, the highly aggressive (grade III) T24 cells were characterized by a prominent efficiency in motility, being able to successfully "heal the wound" in an incubation period of 24 hours, creating a compact monolayer of cells (Figure 10B, panels ix and x). Although administration of 10 μΜ 17-AAG was not able to abrogate T24 proliferation and motility responses, it is clear that the scratch-wound "healing" mechanism in these cells has been significantly impaired due to the effect of the drug, since cells appeared to maintain the gap without being tightly condensed as initially observed under control conditions (Figure 10B, panels xi and xii).

## Discussion

Human urinary bladder cancer is considered an increasingly significant public health issue in the industrialized countries, with a worldwide estimate of about two million patients [2]. Due to the importance of Hsp90 molecular chaperone on client protein maturation and function, along with its voluminous and highly diverse clientele of cancer-related proteins, a variety of Hsp90 inhibitors have emerged as promising anticancer agents [6,12]. In the present study, we have comparatively examined the effects of 17-AAG-induced Hsp90 inhibition on multiple protein targets implicated in signaling pathways critically regulating cell proliferation, apoptosis and motility, in RT4 (grade I), RT112 (grade I-II) and T24 (grade III) human urothelial bladder cancer cells. The data presented herein clearly demonstrate that, upon 17-AAG treatment, cell type-specific downregulation of multiple signaling molecules is followed by cell cycle arrest, finally resulting in Caspase-mediated cell death.

Depending on the cellular context and malignancy grade, 17-AAG has been shown to facilitate arrest in all checkpoints of the cell cycle, as for example, in human malignant pleural mesothelioma (G1 or G2/M block) [20] and breast cancer cells (G1 block) overexpressing HER2 [21]. In all human bladder cancer cell lines examined in this study, apoptotic cell death was found to be preceded predominantly by a drug dose-dependent G1/S cell cycle block, with arrest in other phases of the cell cycle appearing in a cell type-specific manner. The unpredictability of cell cycle arrest induced by 17-AAG in bladder cancer cells is in agreement with previous reports and might be related to differences in client protein repertoires and cellular contexts [22]. To elucidate the 17-AAG-induced block of the cell cycle, we undertook analysis of expression and/or activation profiles of several key-modulators of cell cycle progression. This demonstrated that, in response to 17-AAG exposure, the drug-dependent protein downregulation patterns correlate well with the observed G1 arrest of the cell cycle, as well as with the reduction in cell proliferation capacity.

Implementation of apoptosis, due to the effect of 17-AAG, has previously been reported in glioblastoma [23] and colon cancer [24]. In the bladder cancer cell lines used in this study, cell type-specific and drug dose-dependent activation of a Caspase-induced cell death program proved to be initiated upon 17-AAG administration. These findings are in accordance with the survival rates observed in the cytotoxicity tests, although, in these experiments, 17-AAG-induced cell death percentages in the three bladder cancer cell lines were not found to differ significantly. In contrast, the cell-type specific profile of Caspase repertoire activation, and especially the diminished levels of processed Caspase-3 in RT112 and T24 cells, could possibly implicate other types of executive Caspases not studied here (i.e. Caspase-6 or -7) or even Caspase-independent cell death mechanisms such as autophagy [25,26].

Hsp90 expression levels seem to be upregulated in cancer, resulting in addiction of tumor cells to multiple oncogenic pathways in which Hsp90 clients play a critical role. In bladder cancer, Hsp90 was found to be expressed in more than 90% of human tumor specimens, with high-grade and muscle invasive tumors expressing significantly higher levels of Hsp90 than low-grade and superficial tumours [27]. Nevertheless, in 10% of the tumor samples Hsp90 expression was found to be downregulated and this was associated with infiltrating recurrences and poor prognosis [28,29], most likely due to the overall molecular profile of the individual tumors. Besides the importance of Hsp90 expression levels, specific conformations of the chaperone have been implicated in cancer versus normal cell sensitivity to Hsp90 inhibitors: Hsp90 was shown to display higher binding affinity for 17-AAG exclusively in cancer cells [30], leading to the formation of 17-AAG-sensitive Hsp90-containing "superchaperone" complexes in malignant cells, whereas normal cells bearing a predominantly uncomplexed Hsp90 are significantly less sensitive to these types of inhibitors [13,30]. This feature is likely exploited by Hsp90 targeting with the use of 17-AAG and subsequent effects on multiple Hsp90 targets.

Hsp90 inhibition and subsequent Hsp70 and Hsp27 upregulation, due to 17-AAG, have been reported in human colon [31], prostate [32] and cervical cancer cells [32]. As presented in this study, even though a 17-AAG-induced Hsp90 downregulation was detected in all bladder cancer cell lines over a 24-hours treatment period, a cell type-specific pattern of inhibition was observed. In RT4 and RT112 cells, after exposure to the highest dose of the drug, an additional protein band was generated, whereas no such band could be detected in T24 cells. This novel finding in relation to Hsp90 structural integrity, upon high dose of 17-AAG administration, is presented herein for the first time. We suggest that this fragment may well be a product of Hsp90 proteolytic processing by Granzyme B [14,15]. Use of the GrabCas algorithm has revealed a putative Granzyme B recognition and cleavage site in the amino acid sequence of both Hsp90α and Hsp90β protein isoforms, indicating that Hsp90 must be a *bona fide *substrate of Granzyme B. On the contrary, no Caspase cleavage site could be identified, with the help of GrabCas, fitting to the molecular weight of the possible Hsp90 cleavage fragment under discussion. Interestingly, Hsp90 cleavage has been reported previously, as a response to oxidative stress factors [33], arsenic-based compounds [34] and exposure to doxorubicin and cisplatin chemotherapeutic agents [14,15]. Yet, it is not known whether the putative cleavage product is associated, somehow, with malignancy grade or *p53 *genetic status of the cells, since RT4 and RT112 are grade I and I-II, respectively, harboring a wild-type p53, whereas T24 are grade III, bearing a mutant p53 (Figure 6A). Intriguingly, the RT4- and RT112-specific production of a ~ 65 kDa putative proteolytic fragment could further enhance the functional amputation effect of 17-AAG on Hsp90, likely acting as a putative dominant negative component able to severely impair Hsp90 chaperoning properties. Thus, despite the Hsp90 upregulation observed in response to the highest 17-AAG concentration in grade I (RT4) and I-II (RT112) cell lines, the protein, due to its functional titration by the ~ 65 kDa processed product, seems unable to support its numerous clients thoroughly analyzed here. Therefore, we suggest that the chaperosomes containing these Hsp90 truncated forms are most likely inefficient to exert their cellular tasks.

The three bladder cancer cell lines seemed to follow a distinct and cell type-dependent downregulation profile of the Hsp90 molecular chaperone. However, as shown herein, despite 17-AAG administration, gene expression at the level of transcription remained unaffected for both isoforms of Hsp90 (α and β), clearly indicating that the regulation of Hsp90 is beyond transcriptional control, but occurs more likely at the post-translational level, via ubiquitination and subsequent proteasomal degradation or autophagy.

Hsp90 inhibition was suggested to be tightly associated with a compensatory upregulation of Hsp70 [31] and/or Hsp27 protein levels, likely inducing resistance to 17-AAG [32]. In this work, upon exposure to 17-AAG, total Hsp70 expression levels proved to exhibit a dose-dependent increase and generation of an ~ 65 kDa protein fragment in all three cell lines, reaching peak value at dose 10 μΜ. Using the GrabCas software, we propose that, similarly to Hsp90, the lower molecular weight band could likely represent a product derived from Hsp70 proteolytic processing by 17-AAG-induced Granzyme B activity, but not Caspase protease function.

CHIP was studied in order to illuminate the intriguing pattern of Hsp90 protein level alterations after 17-AAG treatment. CHIP levels were found to be downregulated in a dose-dependent manner in all three bladder cancer cell lines, suggesting a CHIP-regulated effect on proteasomal degradation of associated target proteins, such as Hsp90 and its clients. However, the higher dose-dependent upregulation of Hsp90 and α-tubulin implies a likely redundant, or non-essential, role of CHIP and, therefore, other ubiquitin ligases must be critically implicated in this type of response. An alternative scenario is that affinity threshold phenomena are at play here, with CHIP, although downregulated, still being able to implement its ubiquitin ligase activities regarding Hsp90 clients, but not Hsp90 itself.

The critical role of IGF-IR/Akt signaling pathway deregulation in tumor cell proliferation, survival and migration has been well documented [35]. It has been previously reported that 17-AAG administration causes severe inhibition of the Akt-dependent signaling pathways in osteosarcoma [36] and gastric cancer [37]. As demonstrated here, in human urinary bladder cancer cells, 17-AAG-induced inhibition of Hsp90 resulted in a cell type-specific downregulation of several proteins involved in Akt-dependent signaling, critically contributing to the negative regulation of proliferation, survival and motility. As a consequence, NF-κB transcription null activation potential was significantly compromised, mainly due to the sequestration of the factor into the cytoplasm, as clearly illustrated in Figure 8A. Reduced NF-κB activity was indirectly assessed by measuring the mRNA expression levels of *Survivin *and *cIAP1*, two well known *bona fide *NF-κB target genes. Thus, we have demonstrated that 17-AAG-dependent inhibition of NF-κB activity is tightly associated with transcriptional repression of *Survivin *and *cIAP1 *anti-apoptotic genes, thus decisively contributing to the cytotoxic potency of 17-AAG by decreasing the required "apoptotic threshold" in bladder cancer cells [38].

Moreover, 17-AAG-mediated Hsp90 inhibition resulted in alterations of the phosphorylation status of members of the Forkhead family of transcription factors (FOXO), immediate downstream substrates of Akt kinase, in bladder cancer cells. As shown in this study, FOXO factors proved to be strongly phosphorylated in the highly malignant T24 cells, whereas extremely low, but detectable, levels were also observed in RT112 cells. Administration of 17-AAG caused a notable downregulation of phosphorylated FOXO1 and FOXO3 family members, likely inducing an enhancement of their apoptotic activity.

Interestingly, the undetectable phosphorylation of the IGF-I-dependent downstream mediators (i.e. IGF-IR, Akt, IKKs and FOXOs) in RT4 cells strongly suggests the deactivated character of the pathway under the particular growth conditions, whereas, on the contrary, in T24 cells the IGF-IR/Akt pathway seems to be constitutively activated. RT112 cells proved to display an intermediate pattern of signaling potency, with the IGF-IR/Akt pathway being activated at very low levels. This novel finding of cell type-specific activation of the IGF-IR/Akt-dependent signaling repertoire, herein demonstrated for the first time, could be tightly associated with the underlying differences in various features of the malignant phenotype observed in the three bladder cancer cell lines examined.

Hsp90 inhibition and ensuing Akt inactivation in bladder cancer cells was accompanied by downregulation of Erk1/2-dependent signaling. Exposure to 17-AAG has been previously reported to cause inhibition of the Raf/MEK/ERK signaling cascade in Hodgkin's lymphoma [39] and leukemia [40]. Although total Erk1/2 protein levels exhibited a cell type-specific and drug dose-dependent response similar to the one of α-tubulin and Hsp90, phosphorylated p44/42 levels were severely downregulated in all bladder cancer cell lines, implying the differential control between total and phosphorylated protein destabilization processes in response to the high drug dose treatments.

Invasion and metastasis are one of the hallmark traits of cancer involved in the advanced stages of tumor progression. Hsp90 inhibition by ansamycins has been reported to suppress cancer cell motility and invasion through depletion of the HGF/c-Met signaling pathway in both leiomyosarcoma and glioblastoma cell lines [41]. Another novel finding of the present study is the notable expression and constitutive activation of c-Met receptor in T24 bladder cancer cells, whereas in RT4 and RT112 cells total c-Met protein levels were either absent (RT4) or barely detectable (RT112). Finally, in this study, we have clearly demonstrated that T24 cell line expresses high amounts of phosphorylated IGF-IR, Akt, FOXOs, p44/42 and c-Met proteins and exhibits strong migration dynamics, which could well be associated with a more invasive and metastatic potency, exactly as a result of this over-activated signaling network. Nevertheless, we have shown that the inhibitory effect of 17-AAG on T24 cells is reflected on the significant decrease of both total and phosphorylated c-Met protein levels, with subsequent suppression of other oncogenic parameters, such as increased cell proliferation and motility, hence critically contributing to the impairment of aggressive cancer cell phenotype [41-43].

## Conclusions

**Figure 11:**
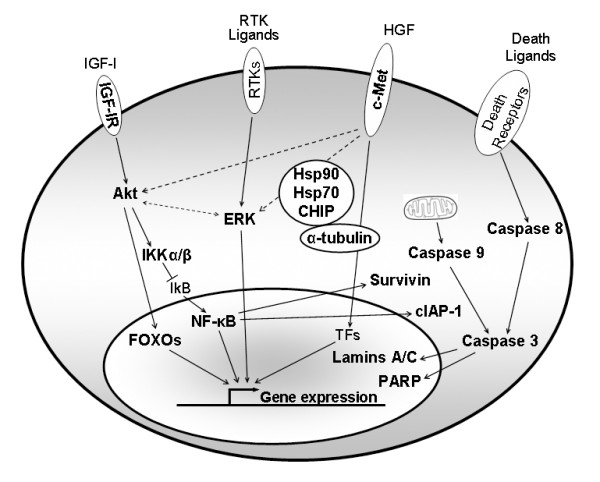
**Schematic representation of the effects of Hsp90 inhibition on multiple signaling pathways in human urinary bladder cancer cells**. Downregulated molecules examined herein are shown in bold letters, while dashed arrows depict "cross-talk" interactions (RTKs: Receptor Tyrosine Kinases, TFs: Transcription Factors).

We have clearly demonstrated the existence of a dose-dependent and cell type-specific inhibitory effect of 17-AAG on cell proliferation, survival and motility in human urinary bladder cancer cells. These responses are likely induced by the pronounced downregulation of multiple Hsp90 protein clients, as well as their associated and downstream components, such as Cyclin D1, Cdk4, pRb, E2F1, IGF-IR, Akt, FOXOs, IKKs, NF-κB, cIAP1, Survivin, ERK1/2 and c-Met, resulting in cell cycle arrest, decline in cell motility and potent activation of Caspase-mediated apoptosis (Figure 11).

## Competing interests

The authors declare that they have no competing interests.

## Authors' contributions

PKK carried out most of the experimental work and drafted the manuscript. DJS has carried out confocal microscopy, participated in the design of the study and helped to draft the manuscript. LHM participated in the design of the study. GEV conceived the study, designed the primers for RT-PCR, participated in the design and coordination of the study and helped to draft the manuscript. All authors read and approved the final manuscript.

## Pre-publication history

The pre-publication history for this paper can be accessed here:

http://www.biomedcentral.com/1471-2407/10/481/prepub
